# Implementation of a team-teaching seminar on the stigmatization and psychosocial burdens of people with visible skin diseases in the standard curriculum of medical studies

**DOI:** 10.3205/zma001774

**Published:** 2025-09-15

**Authors:** Ines Heinen, Rachel Sommer, Inga Hansen-Abeck, Christine Blome, Isabel Heidrich, Martin Härter, Matthias Augustin, Stefan W. Schneider, Finn Abeck, Nina Booken

**Affiliations:** 1 University Medical Center Hamburg-Eppendorf, Hamburg, Germany; 2 University Medical Center Hamburg-Eppendorf, Institute for Health Services Research in Dermatology and Nursing (IVDP), Hamburg, Germany; 3 University Medical Center Hamburg-Eppendorf, Department of Dermatology and Venereology, Hamburg, Germany

**Keywords:** stigmatization, visible skin diseases, dermatology, psoriasis, psychosocial stress, team teaching, interdisciplinary teaching, Kern cycle, curriculum development

## Abstract

**Background:**

Stigmatization of people with visible skin diseases (VSD) is a widespread problem that can lead to severe psychosocial stress. Owing to its great significance, there is also a need to develop and evaluate teaching opportunities for medical students to address stigmatization and the resulting negative consequences.

**Project description:**

The development of the seminar was based on the 6-step Kern cycle. Through the collaboration of dermatologists, psychologists, and an expert on stigmatization in psoriasis, a team-teaching seminar concept on the stigmatization and psychosocial burden of people with psoriasis was developed and implemented in standard teaching at the University Medical Center Hamburg-Eppendorf.

**Results:**

The seminar was held 19 times from October 2023 to July 2024; it was attended by 315 students. Participation in the voluntary questionnaire survey during the seminar was 93% (*n*=293). Data from 284 students were analyzed. This revealed an increase in self-reported knowledge regarding psychosocial stress and stigma among people with VSD. The students rated their ability to advise and support patients who had experienced stigmatization after attending the seminar as higher. Among the students who attended the seminar, 95% were satisfied with it; the majority rated team teaching as useful and the implementation of interdisciplinary teaching as successful.

**Discussion:**

The seminar we designed closes the gap in the standard teaching of clinical and psychosocial subjects on the topic of stigmatization of VSD. By combining different teaching methods (frontal teaching, small group exercises, and discussions) with interdisciplinary team teaching, the sensitive topic of stigmatization in VSD was examined from dermatological and psychological perspectives. Appropriate teaching opportunities for students can improve their medical competence regarding stigmatization and psychosocial stress in people with VSD.

## Zusammenfassung

**Hintergrund:**

Stigmatisierung von Menschen mit sichtbaren Hauterkrankungen (SH) ist ein weitverbreitetes Problem und kann zu schweren psychosozialen Belastungen führen. Aufgrund der hohen Bedeutung besteht auch für die Ausbildung von Medizinstudierenden die Notwendigkeit der Entwicklung und Bewertung von Lehrangeboten, um die Stigmatisierung und hierausfolgende negative Folgen zu thematisieren.

**Projektbeschreibung:**

Die Entwicklung des Seminars erfolgte in Anlehnung an den 6-schrittigen Kern-Zyklus. Durch die Zusammenarbeit von Dermatolog:innen, Psycholog:innen und einer Expert:in für Stigmatisierung bei Psoriasis wurde ein Team-Teaching-Seminarkonzept zur Stigmatisierung und psychosozialen Belastungen von Menschen mit Psoriasis vulgaris entwickelt und am Universitätsklinikum Hamburg-Eppendorf in der Regellehre implementiert.

**Ergebnisse:**

Das Seminar wurde von Oktober 2023 bis Juli 2024 insgesamt 19 Mal durchgeführt und von 315 Studierenden besucht. Die Beteiligung an der freiwilligen Fragebogenerhebung während des Seminars lag bei 93% (*n*=293). Insgesamt konnten die Angaben von 284 Studierenden ausgewertet werden. Dabei ließ sich eine Zunahme des selbstberichteten Wissens über psychosoziale Belastungen sowie Stigma von Menschen mit SH feststellen. Die Studierenden schätzten die eigenen Fähigkeiten, Patient:innen, die Stigmatisierung erfahren haben, zu beraten und zu unterstützen, nach dem Seminarbesuch als höher ein. Mit dem Seminar waren 95% der Studierenden zufrieden, das Team-Teaching wurde von der Mehrheit der Studierenden als nützlich bewertet, auch die Umsetzung des interdisziplinären Unterrichts sei gut gelungen.

**Diskussion:**

Das von uns konzipierte Seminar schließt eine Lücke in der Regellehre von klinischen und psychosozialen Fächern zum Thema der Stigmatisierung von SH. Durch die Verknüpfung unterschiedlicher Lehrmethoden (Frontalunterricht, Übung in der Kleingruppe und Diskussion) mit dem interdisziplinären Team-Teaching wurde das sensible Thema Stigmatisierung bei SH aus dermatologischer und psychologischer Sicht beleuchtet. Geeignete Lehrangebote für Studierende können die ärztliche Kompetenz in Bezug auf Stigmatisierung und psychosoziale Belastungen bei Menschen mit SH verbessern.

## 1. Introduction

Awareness of stigmatization in dermatology has increased, particularly as a result of the 2014 World Health Assembly (WHA) resolution [1]. At that time, the WHA called on its member states to improve the quality of care for people with psoriasis and to explicitly do more to combat stigmatization of those affected [2]. Comprehensive data on social and self-stigmatization were subsequently collected in Germany [3], [4], [5], showing that stigmatization is widespread in Germany as well as worldwide [6], [7]. For patients, the experience of stigmatization and the resulting stress can contribute to anxiety, depression, and impaired quality of life [8]. Patients with psoriasis showed a higher likelihood of developing depressive symptoms and a higher prevalence of anxiety symptoms than those without psoriasis [9]. Stigmatization is considered the most important predictor of depressive symptoms in patients with psoriasis [10]. A large study in 17 European countries also showed that people with skin diseases experience stigmatization more frequently than people with healthy skin. In addition to psoriasis, patients with atopic dermatitis, alopecia, and bullous skin diseases have also reported experiencing stigmatization [11]. Psychosocial stress can be extremely significant; it influences the lives of people with visible skin disease (VSD). According to a survey of patients with psoriasis, 58% stated that the disease had influenced their career choice. These cumulative life-course impairments can significantly affect well-being [12], [13]. Because of the increased risk of mental comorbidities in patients with VSD, early screening is important [8]. Therefore, psychological and social aspects should also be addressed as part of person-centered treatment [14]. Early psychosocial interventions may be necessary to prevent mental stress from becoming chronic in people with VSD [3]. 

Against this background, physicians should be made aware of the importance of stigmatization in patients with VSD. Better treatment decisions can be made and treatment outcomes can be optimized by considering psychosocial parameters such as depression, anxiety, or quality of life alongside clinical-dermatological measures such as the percentage of skin involvement in VSD [14].

Due to the high significance of stigmatization among people with VSD, there is a need to develop and evaluate measures, such as those called for by the WHA, to avoid stigmatization and its resulting negative effects [1]. Therefore, a multidisciplinary consortium consisting of patient representatives, dermatologists, and psychologists developed a nationwide program to destigmatize people with VSD. Two seminars, one for medical students and one for prospective teachers, were conducted to sensitize both target groups to this topic during their professional activities [15], [16]. However, the seminar on destigmatization for people with VSD developed by Sommer et al. (2022) for medical students has not yet been integrated into standard teaching [15].

The aforementioned WHO resolution lists psoriasis as one of the five non-infectious diseases of public health importance [1]. Psoriasis is one of the most widespread chronic and VSDs, with a prevalence of approximately 2% [17]; therefore, it is a good example of a chronic VSD.

To date, the effects of stigma regarding VSD have not been comprehensively covered in the medical studies curriculum in Germany. This topic has not yet been taught in depth in the integrated medical degree program (iMED) at the University Medical Center Hamburg-Eppendorf (UKE) [18]. As the consequences of psoriasis have both dermatological-somatic and psychosocial aspects, we believe it would make sense to teach this in a multidimensional and multiprofessional manner as part of team teaching. The national competence-based learning objectives catalog for medicine (NKLM) adopted in 2021 [https://nklm.de/zend/menu], which defines not only knowledge and skills but also overarching learning objectives such as attitudes and competencies, states that taboo topics and experiences of stigmatization should be addressed appropriately in the context of medical consultations for psoriasis. In the NKLM, medical interviewing in this area is classified as a complex-level learning objective that should be taught in a multi-professional manner, depending on the training level.

To prepare medical students for medical contact with patients with VSD, a new seminar in the form of team teaching on stigmatization and psychosocial stress of people with VSD was developed within the framework of iMED at the UKE, based on the seminar for medical students described above by Sommer et al. (2022), and implemented in regular teaching at the UKE [15]. Team teaching, also known as coteaching, is defined as the joint teaching of a group of students by at least two teachers from different disciplines or scientific backgrounds [19]. This didactic approach was chosen to deepen the transfer of theoretical knowledge for practical and clinical applications [20], [21]. The aim of the project described here was to develop and implement a multi-professional seminar concept with team teaching on stigmatization in VSDs using the example of psoriasis, as well as to test the feasibility and acceptance of this new offer by the students. 

## 2. Project description

### 2.1. Procedure

**Figure 1:**
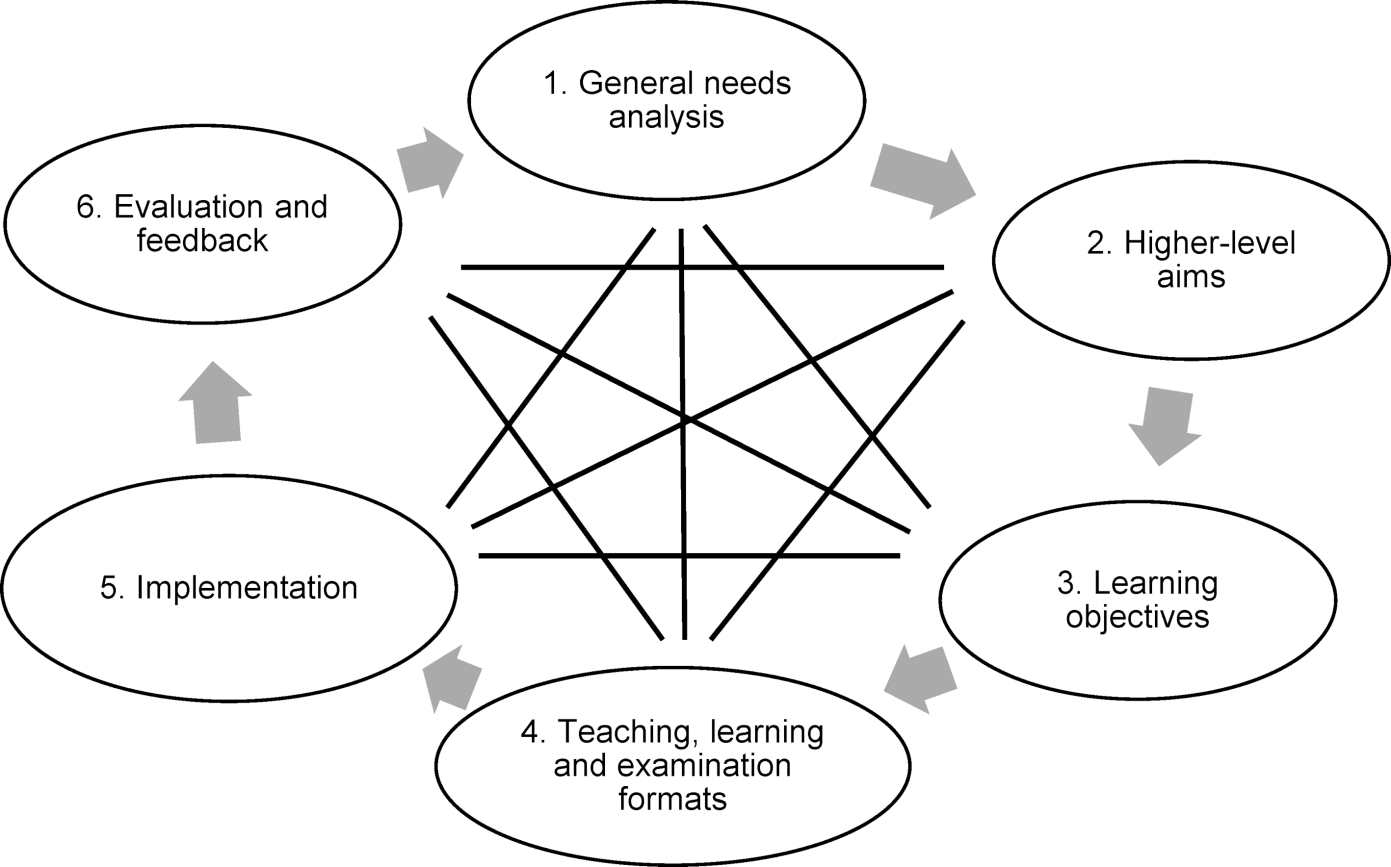
Steps of curriculum development based on the Kern cycle [23]

The seminar was based on the cycle described by Kern et al. (2016) for curriculum development, which consists of six steps: problem definition, needs analysis, goal definition, selection of appropriate methods, implementation, and evaluation (see figure 1 (Fig. 1)) [22]. The Kern cycle was developed for redesigning a curriculum, but can also serve as a basis for individual events, such as the seminar described here. The six steps in the core cycle are to be understood as part of an ongoing process that does not have to be run in a linear fashion, as the methods can be adapted, for example, based on new evaluations. The target group for this project and seminar were medical students in their 6^th^ and 7^th^ semesters who were attending module C3 within the iMED course at the UKE [18]. The steps taken to develop the seminar based on the core cycle (see figure 1 (Fig. 1)) are described below.

### 2.2. Problem definition and general needs analysis 

Based on a WHO publication (2016), a literature review on the topic, and previous work by the working groups involved [1], [3], [4], [5], [15], [16], the necessary competence of students regarding the diagnosis of psoriasis and the diagnosis and treatment of psychosocial stress in VSD, including experiences of stigmatization, were identified as problems. To date, psoriasis has been taught individually, as have medical consultations and the psychosocial burdens of chronic diseases. The stigmatization of VSD has not yet been anchored in curricular teaching across the board. In the iMED course at the UKE [18], medical interviewing is taught at different levels of severity, from basic interviewing skills to participatory decision-making, diversity-sensitive interviewing, and motivational interviewing, by breaking bad news and talking to seriously ill patients and their relatives. In module C3 (6^th^ and 7^th^ semester) of the iMED course, psoriasis was discussed as part of a dermatology lecture. In addition to the seminar presented here, the dermatology department in module C3 held 13 lectures, as well as another seminar and two bedside teaching sessions. This indicates that most dermatology courses took place in this module. Stigma or stigmatization is addressed in the iMED course in the 3^rd^ semester (module F1) by a psychiatrist as part of an introductory lecture on mental or psychiatric illnesses, in module C3 (6^th^ and 7^th^ semester) by medical psychology in a seminar on psychosocial factors in HIV as well as in a lecture and seminar on diversity-sensitive conversation, and in module F2 (also 6^th^ and 7^th^ semester) in a seminar on addiction. To date, there has been no team teaching between dermatology and psychosocial institutions that would make students aware of the need to combine somatic, clinical, and psychosocial communication skills. However, students often fail to integrate input from different subjects into an overall picture if they are not taught together [23]. 

### 2.3. Higher-level aims and learning objectives

The formal aim was to develop a new seminar in Module C3, entitled “psyche and skin” which would combine both the clinical dermatological content related to the diagnosis of psoriasis and the resulting psychosocial stress. Using the example of psoriasis, students were taught in-depth diagnostic knowledge of the disease, as well as background information on common psychosocial impairments. Simultaneously, students should be sensitized to the stigmatizing experiences of patients with VSD. 

The overarching learning objective was to increase students' knowledge and perceived relevance of dermatological and psychosocial problems and needs related to chronic VSD. Furthermore, students' knowledge of the possible stigmatization due to VSD and the psychosocial consequences of such stigmatization was to be increased. The learning objectives were developed based on those of the NKLM for psoriasis [https://nklm.de/zend/menu] (see attachment 1 ).

### 2.4. Teaching, learning, and examination formats

The seminar “psyche and skin” was developed as a compulsory course with attendance recording (group size approximately 20 students each) in the form of team teaching with the participation of the Department for Dermatology and Venerology, the Department for Medical Psychology, and the Institute for Health Services Research in Dermatology and Nursing Professions (IVDP). The course was conducted as part of regular teaching in the 6^th^ and 7^th^ semesters of medical studies in the iMED model course as a compulsory course in module C3 [18]. One lecturer from Dermatology and one lecturer from IVDP, which has a research focus on stigmatization in VSD, were present at each event. In addition to the dermatological content on VSDs with a focus on psoriasis (clinical picture, diagnosis, severity assessment, treatment options and the importance of visible skin areas for treatment decisions [24], [25]) the seminar covered the topics of stigmatization and psychosocial burdens of people with VSD. In addition to psoriasis, the seminar addressed the burden on patients with atopic dermatitis and vitiligo [1], [3], [4], [14]. The students were also taught ways to screen for psychological comorbidities (Health Questionnaire for Patients, PHQ-4 [26]) and an online program to improve well-being and quality of life for people with chronic skin diseases (Skin Compass [https://www.hautkompass.com/de/]). In addition to VSD, stigmatization, and psychosocial stress, the topic of skin diseases in people with skin of color is also covered in this seminar (not part of this publication, see Abeck et al. [27]). The seminar lasted 90 minutes, of which 45 minutes were devoted to teaching about stigmatization and the psychosocial burden of people with VSD.

At the beginning of the seminar, dermatological knowledge was imparted through classic frontal teaching. In addition, a virtual interactive case presentation of a patient with psoriasis was used to illustrate its implementation in everyday clinical practice. The IVDP lecturer then taught the topics of stigmatization and psychosocial stress, initially in the form of traditional frontal teaching, followed by an exercise. In this exercise, the students were divided into small groups and sorted into 16 pictures with different VSD according to their perceived or suspected risk of stigmatization. This exercise was developed by Sommer et al. (2022), and has since been integrated into standard teaching [15]. This was followed by a group discussion wherein the students could present their thoughts on the risk of stigmatization of different VSD.

Currently, the content of the “psyche and skin” seminar is not explicitly tested as part of the multiple choice (MC) exam at the end of module C3. However, aspects of psoriasis can be tested in the MC questions of dermatology, as well as aspects of stigmatization in the MC questions of medical psychology, as the two subjects each have their own courses on these topics in Module C3 (see the above problem definition and general needs analysis).

### 2.5. Implementation

The seminar content was developed by representatives of the Institutes of Dermatology, Medical Psychology, and IVDP in the summer of 2023. After consultation with faculty committees, the seminar was offered for the first time in October 2023 in module C3 for the 7^th^ semester and has been held continuously since then. This project report refers to the winter semester of 2023-24 and the summer semester of 2024.

### 2.6. Evaluation and feedback

During the seminar, students were asked to evaluate and provide feedback on the “psyche and skin” seminar using a written questionnaire handed out by the lecturers at the beginning and collected at the end. At the end of the semester, the students submitted evaluations of the seminar as part of a general online teaching evaluation by the Dean’s Office for Student Affairs. These two evaluation approaches enabled more comprehensive data to be obtained from the students. The online teaching evaluation at the end of the semester has the advantage of being relatively free from the influence of social desirability or lecturer-student interaction. However, evaluations during the seminar allowed more students to be interviewed and more items to be surveyed.

## 3. Results

This was a questionnaire survey in a pre/post design with medical students at the UKE, conducted at 19 compulsory seminars between October 2023 and July 2024. In the iMED model degree program, students must provide proof of participation in at least 85% of the compulsory courses; that is, they can be absent from a maximum of 15% of compulsory courses. This means that, even in a compulsory seminar, attendance of less than 100% is the rule. Before the study began, the study design was reviewed and approved by the local psychological ethics committee at the UKE’s Center for Psychosocial Medicine (LPEK-0688). The students were provided an information sheet on the study prior to the survey. Survey participation was voluntary and anonymous. The students did not receive any compensation for their participation. Nonparticipation in the survey had no negative consequences for students. At the end of the semester, students completed questions about the new team-teaching seminar as part of the general online teaching evaluation by the Dean's Office for Student Affairs. They rated statements such as “Overall, I am satisfied with the seminar” on the Likert scale mentioned above with 6 levels from 1: “strongly disagree” to 6: “strongly agree.”

At the beginning and end of the seminar, students completed a questionnaire. In addition to general student characteristics (age, sex, current semester, previous training/studies, and personal experience with stigmatization and VSD), dermatological knowledge and the relevance of dermatological knowledge about VSDs were assessed, as well as the relevance of knowledge and knowledge of psychosocial stress in patients with VSD, knowledge of the definition of the term stigma, the relevance of stigmatization in patients with VSDs, and their own ability to advise and support patients who have experienced stigmatization according to self-assessment. The assessment was carried out on a Likert scale from 1 to 6 (relevance/knowledge “very low” to “very high”). In addition, at the end of the course, the seminar was evaluated in terms of content and structure (Likert scale from 1: “not at all relevant” to 6: “very relevant”). The 6-point Likert scale used for all items was based on regular teaching evaluations of the medical degree program iMED.

The questionnaire used in the seminar on knowledge and skills as well as the desire for further seminars on this topic consisted of self-formulated items. The items used in the regular teaching evaluation were also used to evaluate seminars and team teaching.

### 3.1. Statistics

Data analyses were performed using GraphPad Prism Software Version 8 (GraphPad Software, San Diego, CA, USA) and IBM SPSS^©^ Statistics version 29. Paired *t*-tests and independent-samples *t*-tests were used to compare knowledge and skill assessments before and after the seminar. The statistical tests were one-sided and the α-level of 5% was set at *p*=0.008 for the paired *t*-tests and at *p*=0.00625 for *t*-tests for independent samples after Bonferroni adjustment for multiple testing. The testing was one-sided, as participation in the seminar was expected to result in an increase in self-assessed competence and knowledge, as well as a higher assessment of the relevance of the teaching content. It was a quasi-experimental pre-post design without a control group or randomization, as the new seminar was introduced to all student groups at the time of module C3 in the winter semester of 2023-24 and in the summer semester of 2024.

### 3.2. Sample 

Of the 394 students in the winter semester of 2023-24 and summer semester of 2024, 315 students attended 19 seminars (80%). As one seminar date in the summer semester fell on a public holiday, and this canceled date was not made up for, 20 students were unable to attend the seminar. Participation in the voluntary questionnaire survey was 93% (*n*=293) of the students in the seminar (*n*=315) and 74% of all students in the two semesters (*n*=394). Nine of the available questionnaires were not included in the analyses, as more than 50% of the sociodemographic questions and questions on stigma and VSD were missing, meaning that *n*=284 questionnaires for the analyses. 

Of the 284 students, 66.9% were female (*n*=190), and 32.7% were male (*n*=93). The mean age was 24.9±3.2 years (range 20-45 years). 40.1% (*n*=114) of the students were in their 6^th^ semester (summer semester, 2024) and 47.5% (*n*=135) were in their 7^th^ semester (winter semester 2023-24). A total of 44.4% (*n*=126) participants stated that they had completed training or studies before starting their medical studies (see table 1 (Tab. 1)).

### 3.3. Previous experiences

In total, 54.2% (*n*=154) of the students stated that they had already experienced stigmatization of their own person at least once during medical contact (see table 2 (Tab. 2)). Sex (32.0%, *n*=91) was the most frequently cited reason for stigmatization according to the students’ self-assessment, followed by migration background (19.4%, *n*=55) and age (9.2%, *n*=26). A total of 88.7% (*n*=185) of the students stated that they knew at least one person with VSD among their friends. In total, 16.2% (*n*=46; see table 2 (Tab. 2)) stated that they were affected by a VSD. A total of 33.5% (*n*=95) of the students reported that they had already witnessed another person being stigmatized because of their skin conditions. Among the respondents, 10.2% (*n*=29; see table 2 (Tab. 2)) reported having been stigmatized due to a skin condition. 

### 3.4. Comparison of self-assessment before and after seminar participation 

Using paired t-tests, the students’ self-assessments of the six items were compared before and after the seminar. The results of the paired t-tests are presented in table 3 (Tab. 3). The effect sizes of Cohen’s d>0.8 indicate a large effect.

The relevance of the knowledge of dermatological problems and needs of patients with VSD was rated by the medical students before the seminar with an average of 5.3±0.8 and afterwards with 5.6±0.6 (Likert scale from 1 “very low relevance” to 6 “very high relevance”). This difference was statistically significant (*t*(257)=-6.776, *p*<0.001). With regard to the students’ own dermatological knowledge of VSD according to self-assessment, there was also a significant increase in knowledge after participation in the seminar (3.6±1.1 vs. 4.8±0.8; *t*(257)=-16.901, *p*<0.001; Likert scale from 1 “very low knowledge” to 6 “very high knowledge”). The students’ self-assessment of their knowledge of psychosocial stress at the start of the seminar averaged 3.9±1.1 (Likert scale from 1 “very low knowledge” to 6 “very high knowledge”). The relevance of knowledge about psychosocial stress in patients with VSD was given an average of 5.3±0.8 before the seminar (Likert scale from 1 “very low relevance” to 6 “very high relevance”). After completing the seminar, the students rated both their knowledge (5.0±0.8; *t*(257)=-14.052, *p*<0.001) and the relevance of psychosocial needs (5.6±0.6; *t*(283)=-6.431, *p*<0.001) significantly higher.

With regard to knowledge about the definition of stigma according to self-assessment, there was a significant increase in knowledge after attending the seminar (4.6±1.1 vs. 5.3±0.7;* t*(283)=-16.058, *p*<0.001; Likert scale from 1 “very low knowledge” to 6 “very high knowledge”). The students also rated the relevance of stigma in patients with VDS significantly higher after completing the seminar (4.6±1.1 vs. 5.5±0.7; *t*(283)=-13.664, *p*<0.001; Likert scale from 1 “very low relevance” to 6 “very high relevance”).

The self-assessed ability to counsel and support patients with VSD who have experienced stigmatization increased significantly from 3.4±1.2 before the seminar to 4.6±0.9 after the seminar (*t*(283)=-17.528, *p*<0.001; Likert scale from 1 “very low ability” to 6 “very high ability”).

### 3.5. Seminar evaluation in class

**Figure 2:**
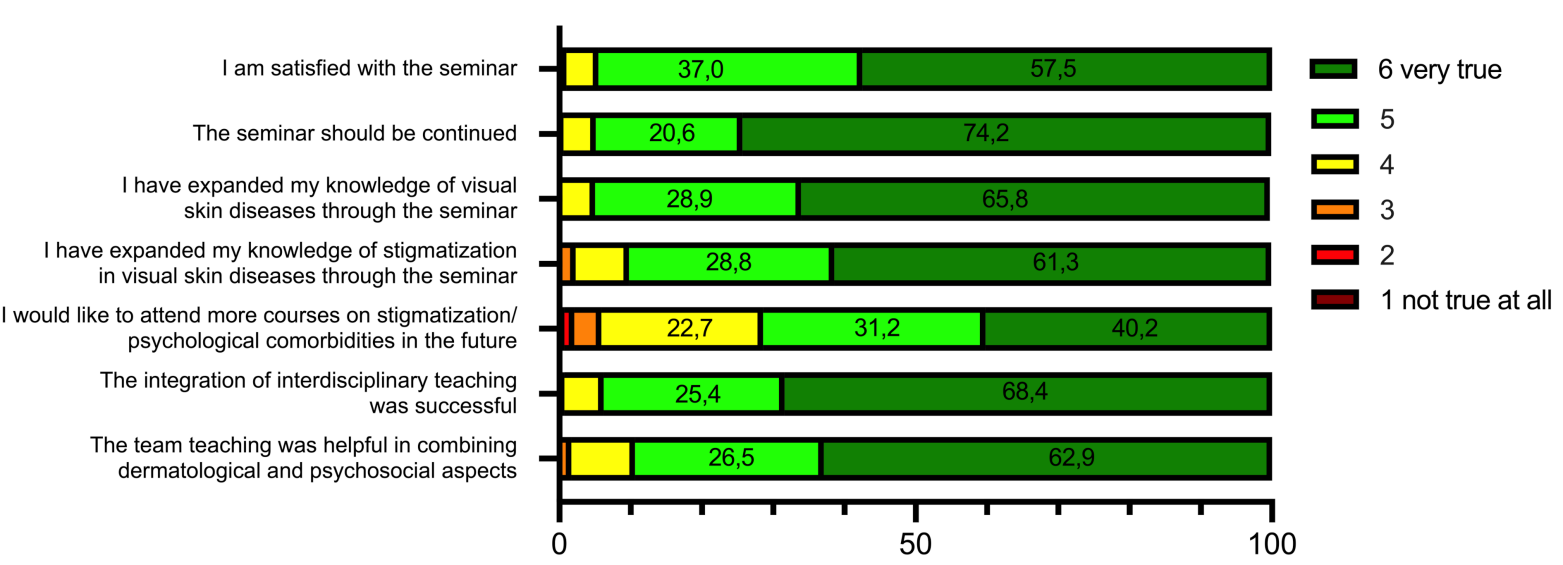
Seminar evaluation by students following the seminar (*n*=258; 6-point Likert scale from 1 “not at all applicable” to 6 “very applicable”), showing the percentage distribution of response.

The majority of students were satisfied with the seminar (94.5% chose 5 or 6 on a scale from 1 “not true at all” to 6 “very true”); 94.7% stated that they had expanded their dermatological knowledge about VSD, and 90.1% stated that they had expanded their knowledge about stigmatization in VSD (each rated 5 or 6 on a 6-point Likert scale). In addition, 71.4% of the respondents stated that they would like to attend more courses on these topics (rating 5 or 6), and 89.4% felt that team teaching was helpful in combining dermatological and psychosocial aspects (rating 5 or 6; see figure 2 (Fig. 2)). 

### 3.6. Group differences in the evaluation of the seminar

To check whether students who are themselves affected by VSD (*n*=46) evaluated the “psyche and skin” seminar differently to students without VSD (*n*=239), *t*-tests were calculated for independent samples. Owing to multiple testing, Bonferroni adjustment was also performed (*p*=0.00625). There were no statistically significant differences between the two groups for any of the items examined (each rated on a Likert scale from 1 “not true at all” to 6 “very true”; see attachment 2 ).

### 3.7. Seminar evaluation at the end of the module

In the general online teaching evaluation of the Dean’s Office for Student Affairs, only the students who took the respective module in the second part of the semester evaluated it; that is, only half of those who attended the module for the entire semester. Therefore, the number of participants in the following results was significantly lower than that of the seminar participants. In this general teaching evaluation, the students each answered two items on the team teaching seminar “psyche and skin” on the previously described Likert scale from 1=“not satisfied” to 6=“very satisfied”. 

In terms of general satisfaction with the course, students in the winter semester 2023-24 rated the “psyche and skin” seminar as the second-best course among all 34 courses in module C3 with a mean value of 5.32 (*n*=73; SD=0.88). In the summer semester of 2024, students rated the “psyche and skin” seminar slightly lower (*n*=85; M=4.91; SD=1.21).

In the winter semester of 2023-24, students rated their satisfaction with the team teaching in the seminar on the connection between theoretical and clinical aspects as 5.45 on average (n=69; SD=0.63). In the summer semester of 2024, the students rated this item with a mean value of 5.15 (*n*=81, SD=1.08).

## 4. Discussion

As the stigmatization of people with skin diseases is a major challenge, the development of intervention options is necessary to protect patients from negative consequences [1], [8], [28]. 

This study investigated the effects of the newly designed “psyche and skin” seminar on stigmatization and psychosocial stress in people with VSD on medical students. Our results show that the self-assessed competence of students on this topic can be improved by offering suitable courses: A significant increase in knowledge about psychosocial stress as well as stigma of people with VSD was reported. The self-assessed ability of medical students to advise and support patients who have experienced stigmatization has also improved. Sommer et al. (2022) investigated a structured intervention for the stigmatization of patients with psoriasis with 127 medical students who had voluntarily registered for the extracurricular course. After participating in the course, the authors observed a reduction in stigmatizing behaviors among medical students [15]. The present study differs from Sommer et al.’s (2022) study in that the primary goal was not to reduce stigmatizing behavior on the part of students, but rather to prepare and train prospective physicians to deal with patients with VSD. In addition, the seminar was implemented as a compulsory course in the standard curriculum. 

To our knowledge, there is currently no compulsory seminar on this topic in the medical curriculum. As the majority of students came into contact with the subject of dermatology as part of their medical curriculum, we considered it sensible to hold seminars on stigmatization and psychosocial stress in people with VSD during their studies. By implementing this as a compulsory seminar, we were able to ensure that most students dealt with these topics. The majority of students expressed a desire for more courses on stigmatization and psychological comorbidities in people with VSD, which we see as a confirmation of our innovative team-teaching concept.

Our evaluation suggests that dermatology knowledge can be improved by offering suitable seminars. However, the care of people with VSD goes far beyond the mere treatment of physical symptoms such as itching. By integrating psychosocial aspects into the treatment process, better treatment decisions can be made and treatment outcomes can be optimized [14]. Therefore, an interdisciplinary team-teaching approach was chosen for this seminar, as both dermatologists and psychologists developed and taught it. Most students rated the seminar as useful and found the implementation of interdisciplinary teaching successful. In addition to greater student involvement in the learning process, the advantages of team teaching include easier linking clinical content to basic knowledge [29]. Team-teaching units can be particularly useful when teaching sensitive topics such as breaking bad news, ethical decision-making – or, as in our case, dealing with stigmatization and psychosocial stress [30]. In our view, the use of the team teaching concept is likely a reason for the high level of student satisfaction with seminars. In the future, further courses on this topic could also address a change of perspective into the role of the patient to be able to derive stigmatization behavior and a reflection on one’s own medical actions. Participation of students and patients in such seminars can also provide valuable input. Owing to its implementation in standard teaching, the teaching time for this complex topic is limited. Extracurricular events could be useful to teach the students in more detail about stigmatization and psychosocial stress in people with VSD.

One of the limitations of this study is the possibility of response bias, for example, due to social desirability or the Hawthorne effect. This cannot be ruled out, particularly in the case of the student-lecturer relationship presented here, as the students completed the questionnaire at the beginning and end of the seminar in the presence of lecturers. However, completion of the questionnaire was voluntary, and non-participation had no negative consequences for the students. Conducting the study as an anonymous survey also helped to reduce social desirability. To further reduce the effects of social desirability on response behavior in student surveys, it could be useful to conduct a survey online or by a third party who is not involved in the course, rather than by a lecturer. Knowledge and competence were assessed based solely on self-assessment. There was no reward for participating in the study. The lecturers did not test the students on the seminar content at any time during the final semester examinations. The quasi-experimental pre-post design without a control group did not allow any causal conclusions to be drawn, as double questioning alone may have resulted in learning effects. Furthermore, this was not a multicenter study, as the seminar was introduced exclusively in the iMED model study program at UKE in Hamburg.

## 5. Conclusion

Suitable courses for students can improve their medical competence regarding stigmatization and psychosocial stress in people with VSD. Interdisciplinary approaches enable a combination of dermatological and psychological aspects that are of great importance in the treatment of patients with VSD. Further courses along similar lines can help train medical students to deal with psychosocial stress in patients and counteract the chronification of accompanying mental illnesses at an early stage.

## Funding

We acknowledge financial support from the Open Access Publication Fund of UKE – Universitätsklinikum Hamburg-Eppendorf.

## Authors

### Last authorship

The authors F. Abeck and N. Booken contributed equally and share last authorship.

### Authors’ ORCIDs

- Ines Heinen: [0000-0001-7361-4554]
- Rachel Sommer: [0000-0002-3080-497X]
- Inga Hansen-Abeck: [0009-0007-5579-4337]
- Christine Blome: [0009-0007-5579-4337]
- Isabel Heidrich: [0000-0001-6894-1913]
- Martin Härter: [0000-0001-7443-9890]
- Matthias Augustin: [0000-0002-4026-8728]
- Stefan W. Schneider: [0000-0002-4679-7137]
- Finn Abeck: [0000-0001-7823-0736]
- Nina Booken: [0009-0005-7692-9240]

## Competing interests

The authors declare that they have no competing interests. 

## Supplementary Material

NKLM 2.0

Supplementary table

## Figures and Tables

**Table 1:**
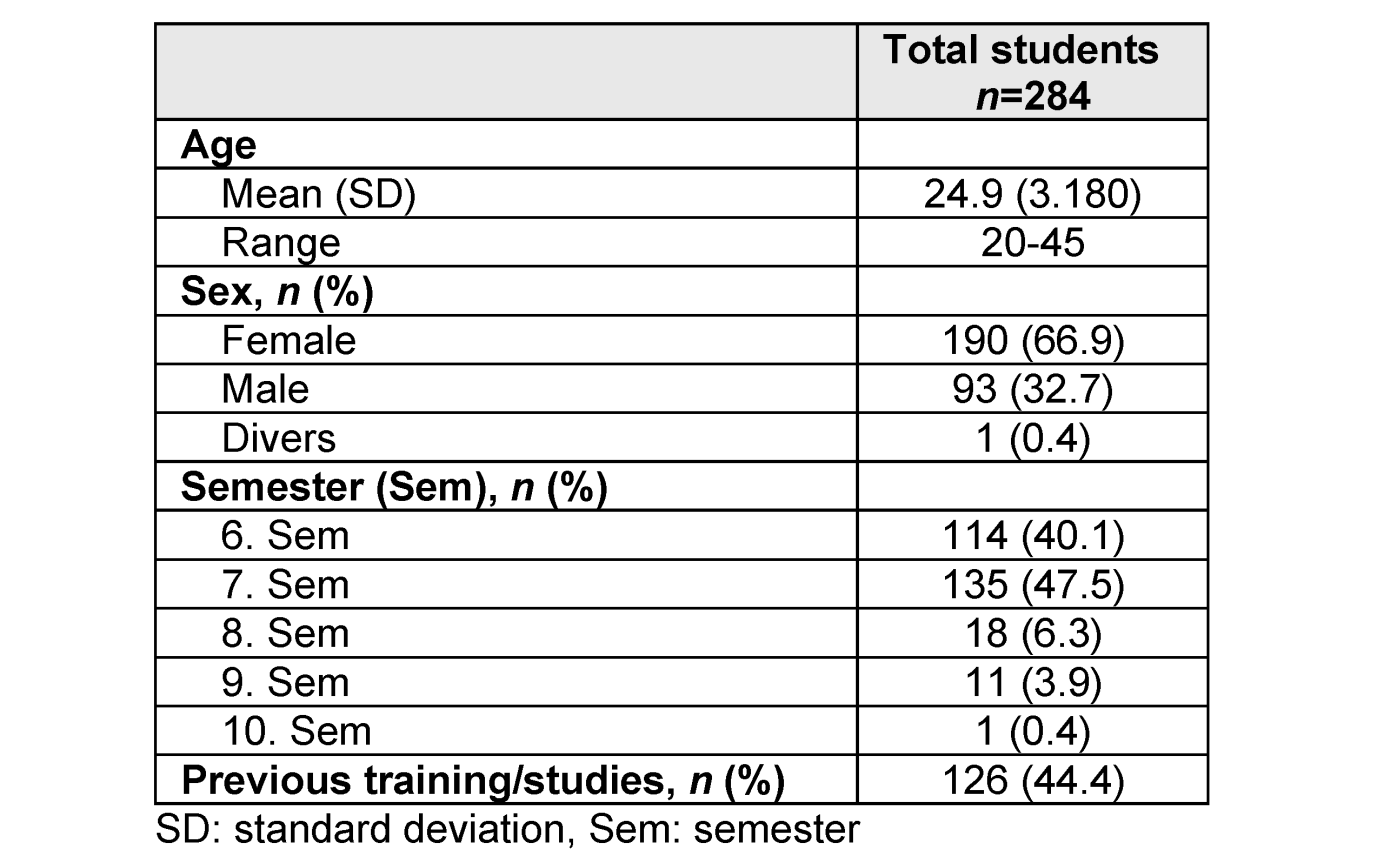
General student characteristics

**Table 2:**
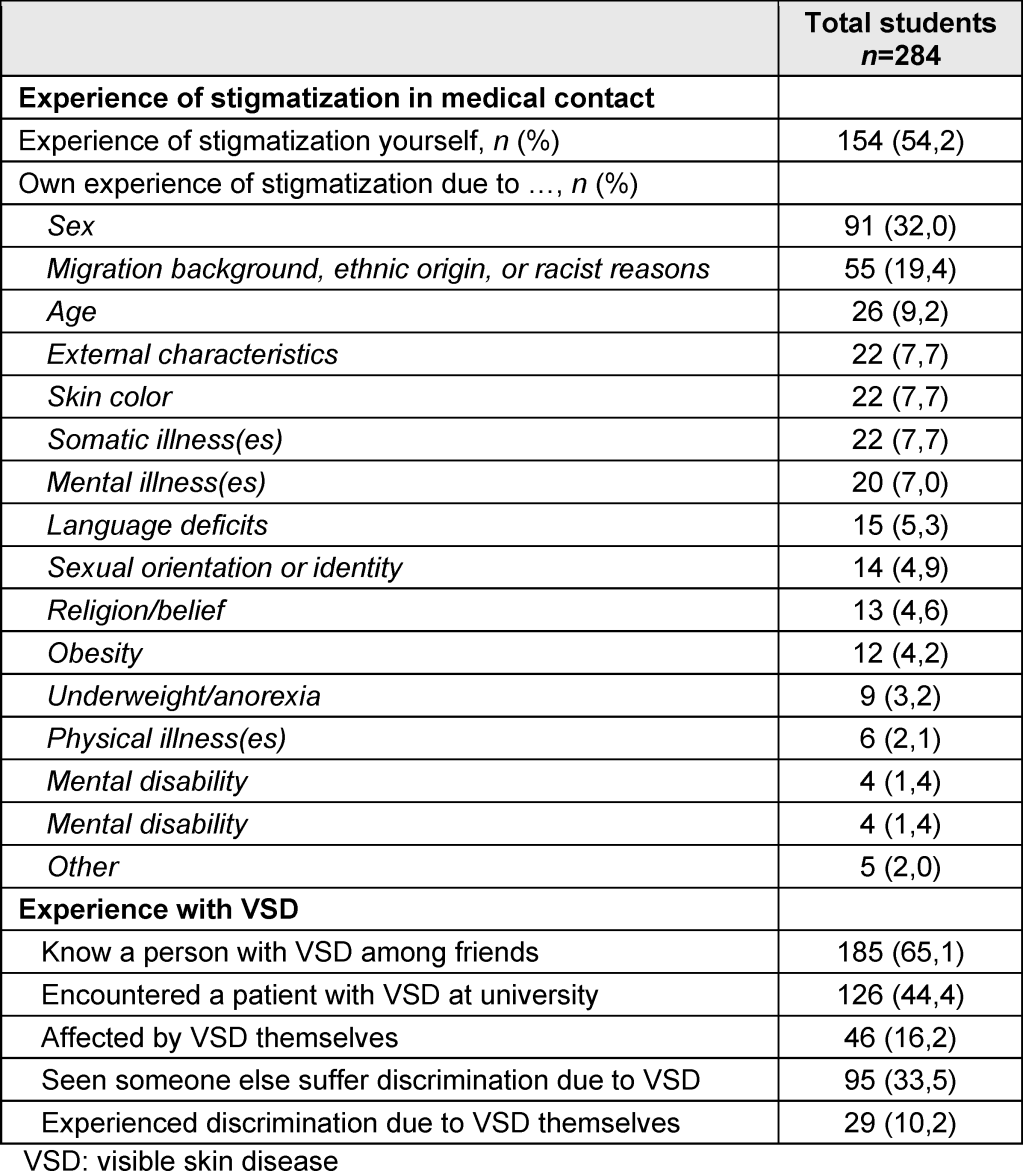
Student experiences with stigmatization and visible skin diseases (VSD)

**Table 3:**
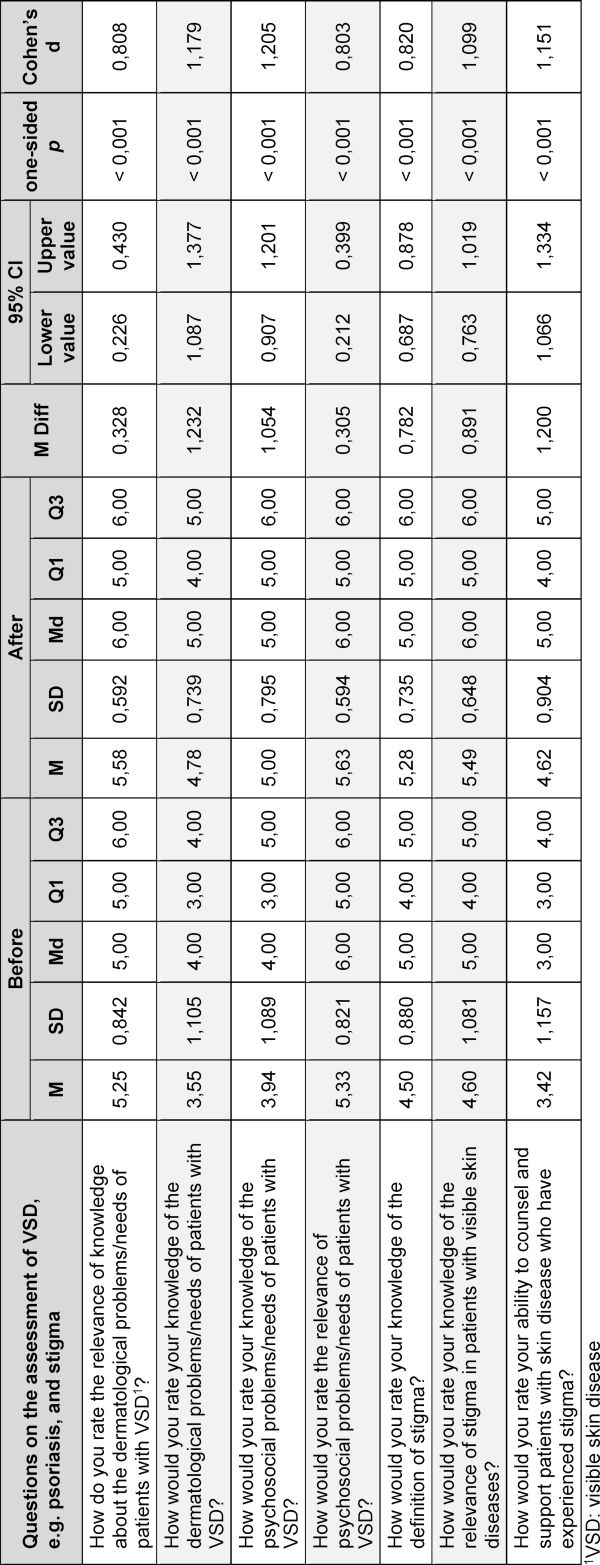
Descriptive data (mean (M), standard deviations (SD), median (Md), percentile 25 (Q1) and percentile 75 (Q3)) as well as information on the paired-samples *t*-tests (mean difference (M diff), 95% confidence interval of the difference (95% CI)) on the self-assessments of the students (*n*=284) before and after seminar participation including effect size Cohen’s d.
