# Supplementary material for: Implementation of a team-teaching seminar on the stigmatization and psychosocial burdens of people with visible skin diseases in the standard curriculum of medical studies
Source: GMS J Med Educ. 2025 Sep 15;42(4):Doc50. doi: 10.3205/zma001774 (PMC12527394; doi:10.3205/zma001774)
Supplement: NKLM 2.0 [file JME-42-50-s-001.pdf]

Attachment 1: NKLM 2.0

Learning objectives on psoriasis vulgaris from the National Competence-based Learning Objectives Catalog for Medicine Version 2.0 (NKLM 2.0) [19]

VI.08-01.1.19 Psoriasis

- VII.1a. Principles of normal structure and function: Explain the interaction of psychological and social factors with the skin and the consequences of skin changes.
- Diagnosis: Students should be able to make a diagnosis based on the clinical picture and medical history and differentiate psoriasis from e.g. eczema; name/trigger psoriasis phenomena, examine joints, differentiate psoriatic arthritis from other rheumatic diseases.
- VII.3 Therapeutic measures:
    - Name and explain the general principles of drug therapy of autoimmune diseases with their drugs.
    - Name the principles of pharmacological treatment of psoriasis with their drugs.
    - Describe the principle of phototherapy and croytherapy and name indications.
    - explain the principles and elements of a participation management plan with the goal-oriented combination of occupational group-specific areas of responsibility.
    - describe concepts of inpatient rehabilitation and apply indication criteria.
    - name the principles of inhalation therapy, phototherapy, Balneo therapy and climatotherapy as well as examples of their methods and indications.
- VIII.2 Conducting medical consultations: perceiving and accepting taboo subjects and stigmatized illnesses and addressing this topic appropriately if it appears useful or necessary.
- VIII.4. health advice, promotion, prevention and rehabilitation: explain the rehabilitation system with its legal basis and the tasks and objectives specific to each institution.
    - On prevention: students should be able to name trigger factors (obesity, infections, mechanical factors, medication, stress, lifestyle)
    - On rehabilitation: indication, information and advice for patients as well as joint decision-making with individual objectives for medical rehabilitation and occupational rehabilitation for working people
- VIII.5 Leadership and management:
    - Name relevant non-medical, supportive care structures/facilities for coping with illness and disability in everyday life and explain their fundamental importance in the context of patient care.
    - Explain basic knowledge of mindfulness methods such as MBSR, apply them independently and also advise patients on them.
    - on management: Students should be able to name the necessity of psychoeducation and the areas that can be contacted in the context of psoriasis treatment (rheumatologists, internists, self-help groups).
- VIII.7 Clinical-practical skills:
    - Carry out a clinical examination of the skin and skin appendages.
    - Ask about, describe and document the patient's understanding of the disease, level of suffering, willingness to change and motivation for therapy.

Attachment 1 to Heinen I, Sommer R, Hansen-Abeck I, Blome C, Heidrich I, Härter M, Augustin M, Schneider SW, Abeck F, Booken N. *Implementation of a team-teaching seminar on the stigmatization and psychosocial burdens of people with visible skin diseases in the standard curriculum of medical studies.* GMS J Med Educ. 2025;42(4):Doc50. DOI: 10.3205/zma001774
