# Supplementary material for: Implementation of a team-teaching seminar on the stigmatization and psychosocial burdens of people with visible skin diseases in the standard curriculum of medical studies
Source: GMS J Med Educ. 2025 Sep 15;42(4):Doc50. doi: 10.3205/zma001774 (PMC12527394; doi:10.3205/zma001774)
Supplement: Supplementary table [file JME-42-50-s-002.pdf]

Attachment 2: Supplementary table

Group differences in the evaluation of the seminar divided into students with a visible skin disease (VSD, *n*=46) and students without VSD (*n*=239); descriptive data (mean (M), standard deviations (SD), median (Md), percentile 25 (Q1) and percentile 75 (Q3)) as well as data on the *t*-tests for independent samples (mean difference (M diff), 95% confidence interval of the difference (95% CI))

Questions on the evaluation of the seminar	students with VSD (*n*=46)					Students without VSD (*n*=239)					M diff	95% KI		one-sided *p*
	M	SD	Md	Q1	Q3	M	SD	Md	Q1	Q3		lower value	upper value	
I am satisfied with the seminar.	5,59	0,541	6,00	5,00	6,00	5,51	0,647	6,00	5,00	6,00	-0,081	-,281	,119	0,214
The seminar should be continued.	5,70	0,511	6,00	5,00	6,00	5,67	0,670	6,00	5,00	6,00	-0,023	-,229	,182	0,411
I have expanded my knowledge of stigmatization in VSD through the seminar.	5,50	0,753	6,00	5,00	6,00	5,49	0,761	6,00	5,00	6,00	-0,015	-,255	,226	0,452
I would like to attend more courses on stigmatization/ psychological comorbidities in the future.	4,87	1,166	5,00	4,00	6,00	5,07	0,976	5,00	4,00	6,00	0,202	-,118	,522	0,108
The integration of interdisciplinary teaching was successful.	5,67	0,560	6,00	5,00	6,00	5,61	0,638	6,00	5,00	6,00	-0,060	-,259	,138	0,275
The team teaching was helpful in combining dermatological and psychosocial aspects.	5,48	0,836	6,00	5,00	6,00	5,52	0,704	6,00	5,00	6,00	0,043	-,188	,273	0,358

[1]VSD: visible skin disease

Attachment 2 to Heinen I, Sommer R, Hansen-Abeck I, Blome C, Heidrich I, Härter M, Augustin M, Schneider SW, Abeck F, Booken N. *Implementation of a team-teaching seminar on the stigmatization and psychosocial burdens of people with visible skin diseases in the standard curriculum of medical studies.* GMS J Med Educ. 2025;42(4):Doc50. DOI: 10.3205/zma001774
